# Predictors of metabolic syndrome in community-dwelling older adults

**DOI:** 10.1371/journal.pone.0206424

**Published:** 2018-10-31

**Authors:** Jeanine M. Van Ancum, Nini H. Jonkman, Natasja M. van Schoor, Emily Tressel, Carel G. M. Meskers, Mirjam Pijnappels, Andrea B. Maier

**Affiliations:** 1 Department of Human Movement Sciences, @AgeAmsterdam, Vrije Universiteit Amsterdam, Amsterdam Movement Sciences, Amsterdam, The Netherlands; 2 Amsterdam Public Health Research Institute, Department of Epidemiology and Biostatistics, VU University Medical Center, Amsterdam, The Netherlands; 3 Department of Rehabilitation Medicine, VU University Medical Center, Amsterdam Movement Sciences, Amsterdam, the Netherlands; 4 Department of Medicine and Aged Care, @AgeMelbourne, Royal Melbourne Hospital, University of Melbourne, Melbourne, Victoria, Australia; Nathan S Kline Institute, UNITED STATES

## Abstract

**Objectives:**

The metabolic syndrome has been associated with a variety of individual variables, including demographics, lifestyle, clinical measures and physical performance. We aimed to identify independent predictors of the prevalence and incidence of metabolic syndrome in a large cohort of older adults.

**Methods:**

The Longitudinal Aging Study Amsterdam is a prospective cohort including community-dwelling adults aged 55–85 years. Metabolic syndrome was defined according to criteria of the National Cholesterol Education Program Adult Treatment Panel III. The incidence of metabolic syndrome was calculated over a period of three years. Stepwise backward logistic regression analyses were used to identify predictors, including variables for demographics, lifestyle, clinical measures and physical performance, both in a cross-sectional cohort (n = 1292) and a longitudinal sub-cohort (n = 218).

**Results:**

Prevalence and incidence of metabolic syndrome were 37% (n = 479) and 30% (n = 66), respectively. Cross-sectionally, heart disease (OR: 1.91, 95% CI: 1.37–2.65), peripheral artery disease (OR: 2.13, 95% CI: 1.32–3.42), diabetes (OR: 4.74, 95% CI: 2.65–8.48), cerebrovascular accident (OR: 1.92, 95% CI: 1.09–3.37), and a higher Body Mass Index (OR: 1.32, 95% CI: 1.26–1.38) were significant independent predictors of metabolic syndrome. Longitudinally, Body Mass Index (OR: 1.16, 95% CI: 1.05–1.27) was an independent predictor of metabolic syndrome.

**Conclusion:**

Four age related diseases and a higher Body Mass Index were the only predictors of metabolic syndrome in the cross-sectional cohort, despite the large variety of variables included in the multivariable analysis. In the longitudinal sub-cohort, a higher Body Mass Index was predictive of developing metabolic syndrome.

## Introduction

Metabolic syndrome (MetS) is defined as the coexistence of abdominal obesity, atherogenic dyslipidaemia, high blood pressure and insulin resistance [1]. MetS is highly prevalent in older adults [2], and has been associated with negative health related outcomes, such as a higher risk of cardiovascular morbidity and mortality [3], decline in mobility [4–6], functional dependence [7], and poorer quality of life [7]. MetS is regarded as a pre-disease state, a warning sign for upcoming multimorbidity [8]. Early risk identification of MetS would allow for targeted interventions to prevent future deterioration of the health status.

Previous studies in adults with a mean age range of 37 to 60 years showed multivariable cross-sectional relations of MetS, including smoking [9, 10], alcohol consumption [10, 11], high body fat levels [12], low socioeconomic status [10, 13], feeling stressed [14] and living with family members [9]. Cross-sectional studies in older adults investigated univariable predictors of MetS, but did not perform multivariable analyses [15–22]. Various variables were predictive of MetS in older adults, including lifestyle and physical performance measures [15–22], which the present study combined in multivariable analyses. Longitudinal studies including adults with a mean age range of 39 to 54 years addressed potential independent predictors of the development of MetS, and revealed age [23, 24], Body Mass Index (BMI) [23–25], waist circumference [26, 27], blood pressure [24, 25], insulin resistance [24, 25, 27] and cholesterol levels [24–27] as predictors of MetS incidence. Predictors for the existence and development of MetS in higher chronological age groups might be different, but evidence is lacking. The prevalence of MetS increases with age [2], and components of MetS differ compared to younger age groups [28], therefore, the present study included a cohort of 65 years and older.

The aim of this study was to identify independent predictors of MetS prevalence in a cross-sectional cohort of community-dwelling older adults and to identify independent predictors of MetS incidence at three years follow-up in a longitudinal sub-cohort. We investigated a large variety of variables, including demographics, lifestyle, clinical measures and physical performance.

## Methods

### Study population

The Longitudinal Aging Study Amsterdam (LASA) is an ongoing longitudinal cohort study with the primary aim to determine predictors, trajectories and consequences of physical, cognitive, emotional and social functioning in relation to ageing. The study started in 1992 in three regions (Zwolle, Oss and Amsterdam) in the Netherlands, and included 3107 community-dwelling participants aged 55–85 years. Participants were recruited from municipal registries, and the oldest old and older men were oversampled. No exclusion criteria were applied. Follow-up assessment cycles are performed every three years, encompassing a main interview with research staff, a medical interview and a self-administered questionnaire. Extended information on the sample and data collection can be found elsewhere [29].

**Fig 1:**
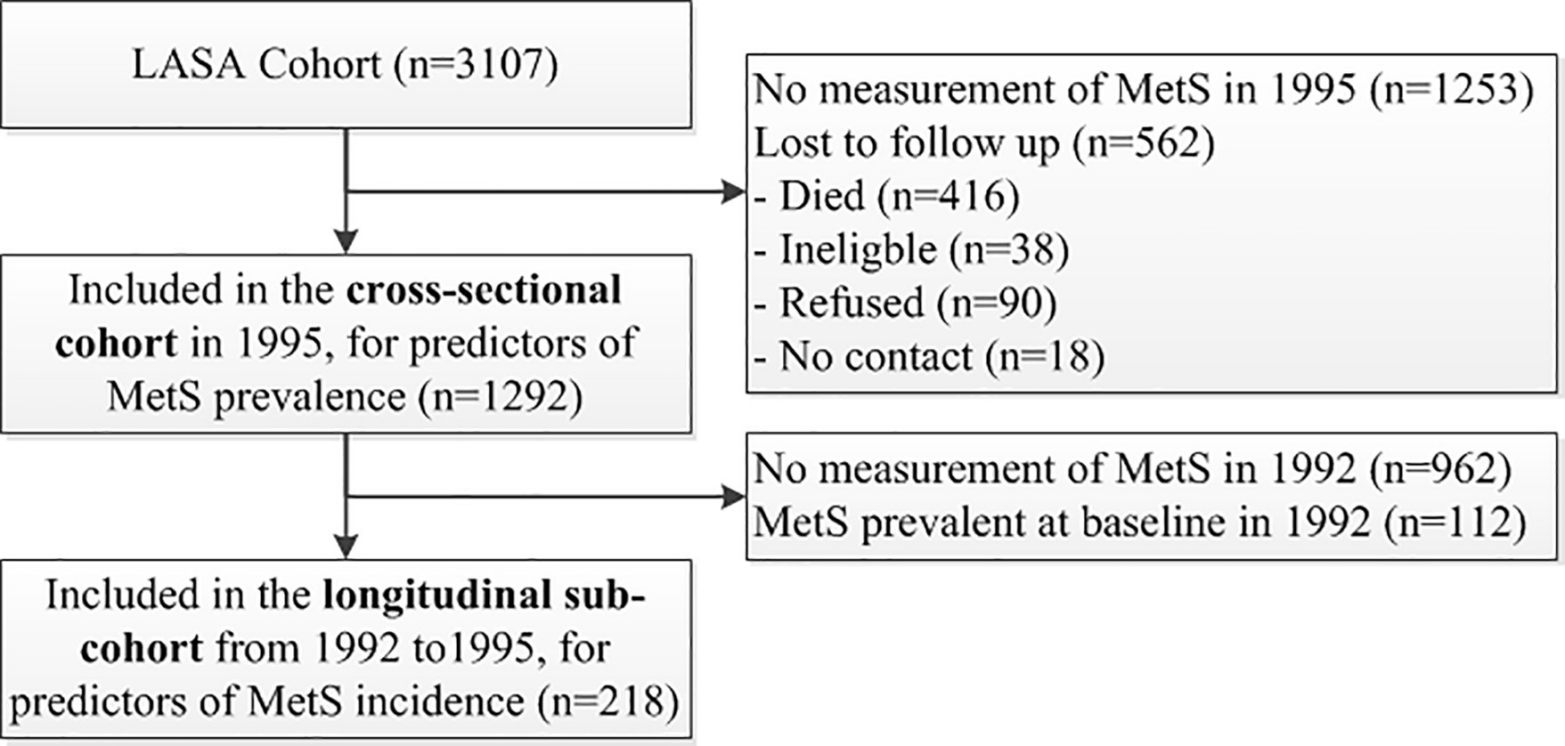
Flowchart. LASA: Longitudinal Aging Study Amsterdam, MetS: Metabolic Syndrome.

In the present study, we included data from the assessment cycles of 1992 and 1995. Blood samples were only obtained from respondents who participated in the medical interview, and who were aged 65 years and older. For the cross-sectional analyses, data of the 1995 cycle (n = 1292) were used as in this cycle all five components of MetS were measured. For the longitudinal analyses, a sub-cohort of the 1992 cycle was used including participants at one of the collaborating centres (Zwolle, the Netherlands). Only participants in whom MetS was not prevalent at baseline in 1992 were included in the longitudinal analyses (n = 218). Fig 1 shows a flowchart of the inclusion of participants. The LASA study was approved by the medical ethics committee of the VU University medical center. All participants signed written informed consent.

### Possible predictors of MetS

Four groups of variables, including demographics, lifestyle, clinical measures and physical performance, were tested as independent predictors of MetS in the cross-sectional and longitudinal analyses.

Demographics included sex, age, living status, educational level, income and occupational status. Sex and age were derived from registries. Living status was assessed by asking participants if they were living alone or not. Educational level was defined as total years of education and divided into three categories: primary education or lower, secondary education and higher education. Income was self-reported and categorized into four groups: low (ƒ<1.750), moderate-low (ƒ1.751–2.250), moderate-high (ƒ2.251–3.500) and high income (ƒ>3.500). Occupational status was assessed by asking participants if they were retired or not.

Lifestyle included smoking status, alcohol consumption and self-reported physical activity. Smoking status was defined as currently smoking or not. Alcohol consumption was reported as number of glasses alcohol consumed per week. The self-reported physical activity was assessed with the LASA Physical Activity Questionnaire (LAPAQ) [30]. The LAPAQ is a validated questionnaire based on seven activities (walking outdoors, bicycling, gardening, light household, heavy household, and up to two sports activities). Participants were asked how often and for how long in the previous two weeks they engaged in each activity. We categorized the score on the LAPAQ into quartiles (quartile 1–4).

Clinical measures composed of cognitive functioning, depressive symptoms, chronic diseases, medication and BMI. Cognitive functioning was measured with the Mini–Mental State Examination (MMSE) [31]. Depressive symptoms were measured with the Centre for Epidemiologic Studies Depression Scale (CES-D) [32]. Chronic diseases were self-reported and included chronic non-specific lung disease, heart disease, peripheral artery disease, diabetes, cerebrovascular accident (CVA), arthritis and cancer. Self-reported medication use was divided into two groups with a cut-off based on polypharmacy, i.e. exceeding four medications [33]. BMI was calculated with height measured to the nearest 0.001 m using a stadiometer, and weight measured to the nearest 0.1 kg using a calibrated bathroom scale, in kg/m^2^.

Physical performance composed of the cardigan test, five-times sit-to-stand (STS) test and gait speed. The cardigan test was assessed with the time in seconds required to put on and take off a cardigan. During the five-times STS test participants were asked to stand up from a sitting position and sit down five times at usual pace with their arms folded across their chest, with time recorded in seconds. Gait speed was measured by recording the time required to walk three meters, to turn around and walk back three meters as quickly as possible and converting this to speed in m/s.

### MetS components

MetS was defined according to the National Cholesterol Education Program Adult Treatment Panel III [1]. The following components were measured in the 1995 cycle: 1) Abdominal obesity (waist circumference >102 cm in men or >88 cm in women) was measured to the nearest 0.1 cm midway between the lower rib margin and the iliac crest following a normal expiration. 2) High triglycerides (≥1.7 mmol/L), 3) low high-density lipoprotein (HDL) (<1.0 mmol/L in men or <1.3 mmol/L in women), and 4) high serum fructosamine (≥247 μmol/L or use of antidiabetic medication). Triglycerides, HDL and fructosamine were determined by an enzymatic colorimetric test (Roche diagnostics, Mannheim, Germany), performed in EDTA plasma samples stored at –80° C at the Department of Clinical Chemistry of the VU University Medical Center (VUmc) in Amsterdam. 5) High blood pressure (≥160/90 mmHg or use of antihypertensive medication) was measured in sitting position using a standard mercury sphygmomanometer (Omron HEM 706) at the upper arm. Participants were considered to have MetS when ≥ three out of five components were present [1].

In the 1992 cycle MetS was based on four components, not including fructosamine. Therefore, at this assessment cycle participants were considered to have MetS when ≥ three out of four components where present. Triglycerides and HDL were determined by the enzymatic colorimetric test (Hitachi 717, CHOD-PAP), performed at the ISALA clinic in Zwolle. Blood pressure was measured using an automatic Omron device (Omron HEM 815F) at the finger. Other components were measured as in the 1995 cycle.

### Statistical analyses

Descriptive statistics of variables were presented as number with % for categorical variables, mean with standard deviation (SD) for normally distributed variables and median with interquartile range (IQR) for skewed variables. Linearity of continuous variables was assessed with logistic regression using dummy variables of quartiles. In case of increasing or decreasing odds ratio’s (OR) in similar direction, the independent variable was assumed to be linearly associated with the outcome [34]. If the linearity assumption was not met, the variable was categorized in tertiles or quartiles.

For the cross-sectional analyses, we performed univariable and multivariable logistic regression analyses. For all univariable analyses, the OR, 95% confidence interval (CI) and p-value (Wald test) were estimated. Variables with a p-value <0.2 in the univariable analyses were selected and included in a multivariable logistic regression. A stepwise backwards selection procedure was applied, until all remaining independent predictors had a p-value <0.05. The explained variance of the multivariable model was reported using Nagelkerke’s R^2^.

The same method was applied in the longitudinal analyses for identifying predictors of MetS incidence at three years follow-up. Selection bias of participants included in the longitudinal sub-cohort (n = 218) compared to the participants without measurement of MetS (n = 962), was investigated using Independent-Samples T Test (normal distribution), Mann-Whitney U test (skewed distribution) or Chi-square test (categorical variables). Differences were considered statistically significant if p<0.05.The Statistical Package for the Social Sciences was used for all analyses (IBM SPSS Statistics for Windows, Version 23.0. Armonk, NY, IBM Corp).

## Results

Detailed characteristics of the cross-sectional cohort and longitudinal sub-cohort can be found in Table 1. The percentage of participants diagnosed with MetS in 1995 was 37% (n = 479). The components of MetS were prevalent in 52% of the participants for abdominal obesity, 31% for high triglycerides, 36% for low HDL, 63% for high blood pressure and 25% for high serum fructosamine. Prevalence of medication use was 5% for antilipaemics, 33% for antihypertensives and 6% for antidiabetics. Of the participants diagnosed with MetS, 58% were female and the mean age was 75.7 (SD 6.6) years. The percentage of participants developing MetS over three years was 30% (n = 66), of which 49% was female and a mean age was 71.2 (SD 6.2) years.

**Table 1:** Characteristics of the cross-sectional cohort and the longitudinal sub-cohort.

Variables			Cross-sectional cohort (n = 1292)			Longitudinal sub-cohort (n = 218)		
			N	MetS prevalence^a^		N	MetS incidence^b^	
				No (n = 813)	Yes (n = 479)		No (n = 152)	Yes (n = 66)
**Demographics**								
	Sex, female		1292	384 (47.2)	278 (58.0)	218	66 (43.4)	32 (48.5)
	Age, years, mean (SD)		1292	75.3 (6.5)	75.7 (6.6)	218	71.3 (6.5)	71.2 (6.2)
	Living status, living alone		1292	308 (37.9)	214 (44.7)	218	38 (25.0)	26 (39.4)
	Education	*Primary*	537	300 (37.0)	237 (49.5)	84	57 (37.5)	27 (40.9)
		*Secondary*	599	399 (49.2)	200 (41.8)	104	73 (48.0)	31 (47.0)
		*Higher*	154	112 (13.8)	42 (8.8)	30	22 (14.5)	8 (12.1)
	Income	*Low*	285	159 (21.0)	126 (29.1)	59	41 (30.1)	18 (30.0)
		*Moderate-low*	286	175 (23.1)	111 (25.6)	37	22 (16.2)	15 (25.0)
		*Moderate-high*	342	219 (29.0)	123 (28.4)	50	35 (25.7)	15 (25.0)
		*High*	276	203 (26.9)	73 (16.9)	50	38 (27.9)	12 (20.0)
	Retired		1259	752 (94.7)	448 (96.3)	217	132 (86.8)	61 (93.8)
**Lifestyle**								
	Current smoking		1292	160 (19.7)	74 (15.4)	218	28 (18.4)	11 (16.7)
	Alcohol, units/week, median (IQR)		1291	4 (0−12)	2 (0−7)	217	3.0 (0−7)	2 (0−7)
	LAPAQ	*Quartile 1*	311	183 (23.3)	128 (28.0)	54	38 (25.2)	16 (24.6)
		*Quartile 2*	312	208 (26.5)	104 (22.8)	54	39 (25.8)	15 (23.1)
		*Quartile 3*	319	196 (24.9)	123 (26.9)	54	34 (22.5)	20 (30.8)
		*Quartile 4*	301	199 (25.3)	102 (22.3)	54	40 (26.5)	14 (21.5)
**Clinical**								
	MMSE, median (IQR)		1289	28 (26−29)	27 (26−29)	218	28 (26−29)	28 (27−29)
	CES-D, median (IQR)		1257	6 (3−12)	6 (2−11)	218	4 (1−10)	5 (2−10)
	Chronic diseases	*Pulmonary disease*	1291	126 (15.5)	64 (13.4)	218	19 (12.5)	8 (12.1)
		*Heart disease*	1291	184 (22.7)	158 (33.0)	218	27 (17.8)	14 (21.2)
		*Peripheral artery disease*	1291	65 (8.0)	75 (15.7)	218	11 (7.2)	7 (10.6)
		*Diabetes*	1291	27 (3.3)	76 (15.9)	218	6 (3.9)	5 (7.6)
		*CVA*	1291	42 (5.2)	56 (11.7)	218	5 (3.3)	2 (3.0)
		*OA or RA*	1291	363 (44.7)	237 (49.5)	218	51 (33.6)	25 (37.9)
		*Cancer*	1291	89 (11.0)	61 (12.7)	218	13 (8.6)	5 (7.6)
	Polypharmacy		1292	98 (12.1)	115 (24.0)	212	5 (3.4)	6 (9.2)
	BMI, kg/m^2^, mean (SD)		1282	25.6 (3.9)	29.0 (3.9)	218	25.2 (3.3)	27.3 (3.9)
**Physical performance**								
	Cardigan test, sec, mean (SD)		1263	13.1 (6.2)	14.2 (7.0)	215	12.0 (5.4)	12.3 (5.6)
	5-times STS test, sec, mean (SD)		1143	13.1 (5.0)	13.9 (5.4)	213	11.9 (3.8)	12.7 (5.7)
	Gait speed, meter/sec, mean (SD)		1234	0.8 (0.3)	0.8 (0.3)	216	1.0 (0.4)	0.9 (0.3)
**MetS components**								
	BP, mmHg, mean (SD)	*Systolic*	1271	149.9 (24.9)	159.4 (26.1)	218	120.6 (22.8)	125.4 (17.7)
		*Diastolic*	1271	82.1 (12.6)	85.7 (14.7)	218	66.6 (14.1)	67.0 (16.2)
	WC, cm, mean (SD)	*Males*	614	96.0 (9.5)	105.2 (8.4)	120	95.5 (8.2)	101.7 (9.4)
		*Females*	627	88.0 (10.7)	98.3 (9.6)	98	94.1 (11.0)	99.7 (12.7)
	Triglycerides, mmol/L, mean (SD)		1291	1.2 (0.5)	2.1 (0.9)	218	1.8 (0.8)	2.1 (1.1)
	HDL, mmol/L, mean (SD)	*Males*	628	1.4 (0.4)	1.0 (0.2)	120	1.3 (0.3)	1.1 (0.3)
		*Females*	659	1.6 (0.4)	1.1 (0.3)	98	1.5 (0.3)	1.4 (0.3)
	LDL, mmol/L, mean (SD)	*Males*	627	3.4 (0.9)	3.7 (1.0)	120	3.7 (1.0)	3.8 (0.9)
		*Females*	652	3.7 (0.8)	4.0 (1.1)	98	4.0 (1.0)	4.3 (1.4)
	Fructosamine, μmol/L, mean (SD)		1289	227.8 (29.9)	248.5 (45.6)		−*	−*

All variables are presented as n (%) unless indicated otherwise. BMI: Body Mass Index, BP: Blood Pressure, CES-D: Centre for Epidemiologic Studies-Depression Scale, CVA: Cerebrovascular Accident, HDL: High-Density Lipoprotein cholesterol, IQR: Interquartile Range, LAPAQ: LASA Physical Activity Questionnaire, MetS: Metabolic Syndrome, MMSE: Mini–Mental State Examination, N: Number of participants, OA: Osteoarthritis, RA: Rheumatoid Arthritis, SD: Standard Deviation, STS: Sit-To-Stand, WC: Waist circumference.

^a^ MetS prevalence: baseline values T1 (1995).

^b^ MetS incidence: baseline values T0 (1992–1995).

* Not reported as this variable was not measured in 1992.

### Cross-sectional analyses

In the univariable analyses, statistically significant associations with Mets were found for female sex, living alone, lower education, lower income, lower alcohol consumption, having a heart disease, peripheral artery disease, diabetes, CVA, osteoarthritis or rheumatoid arthritis, polypharmacy, higher BMI, lower cognition, longer duration of the cardigan test, longer duration of the five-times STS test and lower gait speed (Table 2). The multivariable analysis resulted in five variables to be independent predictors of MetS prevalence in the cross-sectional cohort of community-dwelling older adults: heart disease (OR: 1.91, 95% CI: 1.37–2.65), peripheral artery disease (OR: 2.13, 95% CI: 1.32–3.42), diabetes (OR: 4.74, 95% CI: 2.65–8.48), CVA (OR: 1.92, 95% CI: 1.09–3.37), and a higher BMI (OR: 1.32, 95% CI: 1.26–1.38). The overall explained variance of the multivariable model R^2^ was 31%.

**Table 2:** Potential predictors of MetS prevalence in the cross-sectional cohort of community-dwelling older adults.

Variables			Univariable			Multivariable		
			Odds Ratio	95% CI	p-value	Odds Ratio	95% CI	p-value
**Demographics**								
	Sex, female		1.55	1.23–1.94	<0.001			
	Age, years		1.01	0.99–1.03	0.21			
	Living status, living alone		1.32	1.05–1.67	0.02			
	Education	*Primary vs higher*	2.11	1.42–3.12	<0.001			
		*Secondary vs higher*	1.34	0.90–1.98	0.15			
	Income	*Low vs high*	2.20	1.55–3.14	<0.001			
		*Moderate-low vs high*	1.76	1.23–2.52	<0.01			
		*Moderate-high vs high*	1.56	1.10–2.21	0.01			
	Retired		1.47	0.83–2.62	0.19			
**Lifestyle**								
	Current smoking		0.75	0.55–1.01	0.06			
	Alcohol, units/week		0.98	0.97–0.99	0.001			
	LAPAQ, quartiles	*1 vs 4*	1.37	0.98–1.90	0.06			
		*2 vs 4*	0.98	0.70–1.36	0.89			
		*3 vs 4*	1.22	0.88–1.70	0.23			
**Clinical measures**								
	MMSE		0.95	0.92–0.99	0.01			
	CES-D		1.00	0.98–1.01	0.84			
	Chronic Diseases	*Pulmonary disease*	0.84	0.61–1.16	0.29			
		*Heart disease*	1.68	1.31–2.16	<0.001	1.91	1.37–2.65	<0.001
		*Peripheral artery disease*	2.13	1.50–3.04	<0.001	2.13	1.32–3.42	0.002
		*Diabetes*	5.48	3.48–8.64	<0.001	4.74	2.65–8.48	<0.001
		*CVA*	2.43	1.60–3.68	<0.001	1.92	1.09–3.37	0.024
		*OA or RA*	1.21	0.97–1.52	<0.001			
		*Cancer*	1.19	0.84–1.68	0.34			
	Polypharmacy		2.31	1.71–3.10	<0.001			
	BMI, kg/m^2^		1.26	1.22–1.31	<0.001	1.32	1.26–1.38	<0.001
**Physical performance**								
	Cardigan test, sec		1.03	1.01–1.04	0.01			
	5-times STS test, sec		1.03	1.01–1.06	0.01			
	Gait speed, meter/sec		0.40	0.26–0.63	<0.001			

95% CI: 95% confidence interval, BMI: Body Mass Index, CES-D: Centre for Epidemiologic Studies-Depression Scale, CVA: Cerebrovascular Accident, HDL: High-Density-Lipoprotein, IQR: Interquartile Range, LAPAQ: LASA Physical Activity Questionnaire, MetS: Metabolic Syndrome, MMSE: Mini–Mental State Examination, N: Number of participants, OA: Osteoarthritis, RA: Rheumatoid Arthritis, SD: Standard Deviation, STS: Sit-To-Stand.

### Longitudinal analyses

S1 Table shows the comparison between participants that were included in the longitudinal sub-cohort (n = 218) and the participants without measurement of MetS (n = 962). The longitudinal sub-cohort was younger and healthier, with lower prevalence of polypharmacy and better physical performance, compared to the entire cohort. In the univariable analyses, living alone (OR: 1.95, 95% CI: 1.05–3.61) and a higher BMI (OR: 1.19, 95% CI: 1.09–1.30) were statistically significant predictors of MetS incidence in the longitudinal sub-cohort of community-dwelling older adults (Table 3). In the multivariable analysis, a higher BMI remained a statistically significant predictor (OR: 1.16, 95% CI: 1.05–1.27). The overall explained variance of the multivariable model resulted in an R^2^ of 11%.

**Table 3:** Potential predictors of MetS incidence at three years follow-up in the longitudinal sub-cohort of community-dwelling older adults.

Variables			Univariable		
			Odds Ratio	95% CI	p-value
**Demographics**					
	Sex, female		1.23	069–2.19	0.49
	Age, years		1.00	0.95–1.04	0.91
	Living status, living alone		1.95	1.05–3.61	0.03
	Education	*Primary vs higher*	1.30	0.51–3.30	0.58
		*Secondary vs higher*	1.17	0.47–2.91	0.74
	Income	*Low vs high*	1.39	0.59–3.26	0.45
		*Moderate-low vs high*	2.16	0.86–5.43	0.10
		*Moderate-high vs high*	1.36	0.56–3.30	0.50
	Retired		2.31	0.76–7.05	0.14
**Lifestyle**					
	Current smoking		0.89	0.41–1.91	0.76
	Alcohol, units/week		0.99	0.95–1.03	0.56
	LAPAQ, quartiles	*1 vs 4*	1.20	0.52–2.80	0.67
		*2 vs 4*	1.10	0.47–2.58	0.83
		*3 vs 4*	1.68	0.74–3.82	0.22
**Clinical measures**					
	MMSE		1.11	0.95–1.29	0.20
	CES-D		1.01	0.97–1.05	0.75
	Chronic Diseases	*Pulmonary disease*	0.97	0.40–2.33	0.94
		*Heart disease*	1.25	0.61–2.57	0.55
		*Peripheral artery disease*	1.52	0.56–4.11	0.41
		*Diabetes*	2.00	0.59–6.78	0.27
		*CVA*	0.92	0.17–4.86	0.92
		*OA or RA*	1.21	0.66–2.20	0.54
		*Cancer*	0.88	0.30–2.57	0.81
	Polypharmacy		2.89	0.85–9.83	0.09
	BMI, kg/m^2^		1.19	1.09–1.30	<0.001*
**Physical performance**					
	Cardigan test, sec		1.01	0.96–1.07	0.70
	5-times STS test, sec		1.04	0.98–1.11	0.21
	Gait speed, meter/sec		0.47	0.18–1.20	0.11

95% CI: 95% confidence interval, BMI: Body Mass Index, CES-D: Centre for Epidemiologic Studies-Depression Scale, CVA: Cerebrovascular Accident, HDL: High-Density-Lipoprotein, IQR: Interquartile Range, LAPAQ: LASA Physical Activity Questionnaire, MetS: Metabolic Syndrome, MMSE: Mini–Mental State Examination, N: Number of participants, OA: Osteoarthritis, RA: Rheumatoid Arthritis, SD: Standard Deviation, STS: Sit-To-Stand.

* BMI remains significant after multivariable analysis: Odds 1.16, 95% CI 1.05–1.27, p-value 0.002.

## Discussion

When exploring independent predictors of MetS prevalence in the cross-sectional cohort of community-dwelling older adults, presence of a heart disease, peripheral artery disease, diabetes, CVA and a higher BMI were predictive of MetS. A higher BMI was an independent predictor of MetS incidence at three years follow-up in the longitudinal sub-cohort.

We expected that the predictors of MetS would consist of a variety of variables, including demographics, lifestyle, clinical measures, and physical performance. However, only five predictors of MetS prevalence were identified in the cross-sectional cohort, and only one predictor was identified in the longitudinal cohort. Due to the low number of predictors, the explained variance of the models was low. Prediction of the development of MetS in older adults is complex, which may be due to the multifactorial nature of MetS: MetS consists of multiple components. When applying the diagnostic cut-off of three out of five MetS components to the population, a heterogeneous cohort is composed [35]. This composed population could also explain the complex prediction of MetS, and the relatively low R^2^ observed for both the cross-sectional and longitudinal multivariable models.

We reported a set of common comorbidities, heart disease, peripheral artery disease, diabetes and CVA to be predictors of MetS prevalence in the cross-sectional cohort of community-dwelling older adults. As MetS is a precursor of these comorbidities [3], it is likely that participants first developed MetS and subsequently got diagnosed with the related diseases. This is also supported by our multivariable longitudinal analysis in which the comorbidities were not identified as predictors of MetS incidence.

In the multivariable cross-sectional analysis, a higher BMI remained an independent predictor of MetS. Our findings build upon two previous studies showing in univariable analyses that older adults with MetS have higher levels of BMI [15, 16]. Moreover, a higher BMI as well as obesity are well-known predictors of components of MetS, including hypertension, dyslipidemia and diabetes [36, 37].

In concordance with the cross-sectional analysis, the longitudinal multivariable analysis did not provide multiple predictors of MetS incidence. In fact, only a higher BMI was found to be predictive of MetS, during a follow-up of three years. Applying lifestyle modifications for 12 months has shown to reduce body weight, lower BMI, and subsequently prevent the development of MetS [38]. In line with our results, previous studies in younger adults identified BMI as a predictor of MetS incidence in longitudinal cohorts [23–25]. Since this finding of BMI as a longitudinal independent predictor is supported by our cross-sectional analysis and previous literature [23–25], we would speculate that a higher BMI precedes MetS. This in turn leads to a deterioration of the health status. Previous studies in younger adults identified BMI cut-off values up to 25.5 kg/m2 to be best associated with MetS prevalence [39, 40]. At older age, a shift occurs in the cut-off values for some of the components of MetS [41]. Therefore, the optimal cut-off value for BMI to predict MetS is likely to be higher in older adults, but this should be investigated in larger longitudinal studies. Screening adults with a higher BMI for MetS should be emphasized.

### Strengths and limitations

This is the first study to investigate in a representative sample of community-dwelling older adults a large variety of independent predictors of MetS. However, some limitations are worth discussing. First, we included variables based on questionnaires, like the LAPAQ physical activity questionnaire. Objective physical activity monitoring with accelerometers might have provided a more accurate prediction of MetS. Possible effects of underlying morbidity and medication use could not be eliminated. The prevalence of triglycerides, HDL and fructosamine was based on a single blood assessment, which may have introduced misclassification bias. Furthermore, the longitudinal analyses were limited by a smaller sample size and discrepancies in the sequential measurements of the components of MetS in 1992 and 1995. The sub-cohort was slightly younger and healthier compared to participants not included in the longitudinal analyses. This possible selection bias of participants might have caused an underestimation of MetS incidence after three years follow-up. However, the independent predictor identified in the longitudinal analysis is in line with the multivariable cross-sectional analysis and could provide a relevant component for future screening tools of MetS in older adults. Larger longitudinal studies in older adults are needed to confirm our findings, and prospective population-based studies are needed to investigate the mechanisms behind these findings.

## Conclusion

Cross-sectionally, heart disease, peripheral artery disease, diabetes, CVA and a higher BMI were the only predictors of MetS prevalence in community-dwelling older adults, despite the large variety of variables included in the multivariable analysis. A higher BMI was predictive of MetS incidence at three years follow-up in a longitudinal sub-cohort of older adults. A higher BMI is already recognized as an important risk factor for age related disease in primary care, but should also be used as identifier of older adults at risk for development of MetS.

## Supporting information

S1 Table Characteristics of participants that were included in the longitudinal sub-cohort (n = 218), and participants without measurement of MetS (n = 962). All variables are presented as n (%) unless indicated otherwise. BMI: Body Mass Index, BP: Blood Pressure, CES-D: Centre for Epidemiologic Studies-Depression Scale, CVA: Cerebrovascular Accident, HDL: High-Density Lipoprotein cholesterol, IQR: Interquartile Range, LAPAQ: LASA Physical Activity Questionnaire, MetS: Metabolic Syndrome, MMSE: Mini–Mental State Examination, N: Number of participants, OA: Osteoarthritis, RA: Rheumatoid Arthritis, SD: Standard Deviation, STS: Sit-To-Stand, WC: Waist circumference. * Statistically significant different compared to participants without measurement of MetS (p<0.05).
(DOCX)
