# Supplementary material for: Predictors of metabolic syndrome in community-dwelling older adults
Source: PLoS One. 2018 Oct 31;13(10):e0206424. doi: 10.1371/journal.pone.0206424 (PMC6209297; doi:10.1371/journal.pone.0206424)
Supplement: S1 Table — All variables are presented as n (%) unless indicated otherwise. BMI: Body Mass Index, BP: Blood Pressure, CES-D: Centre for Epidemiologic Studies-Depression Scale, CVA: Cerebrovascular Accident, HDL: High-Density Lipoprotein cholesterol, IQR: Interquartile Range, LAPAQ: LASA Physical Activity Questionnaire, MetS: Metabolic Syndrome, MMSE: Mini–Mental State Examination, N: Number of participants, OA: Osteoarthritis, RA: Rheumatoid Arthritis, SD: Standard Deviation, STS: Sit-To-Stand, WC: Waist circumference. * Statistically significant different compared to participants without measurement of MetS (p<0.05). (DOCX) [file pone.0206424.s001.docx]

| **Variables** | | | | **Without measurement (n=962)** | **Included in sub-cohort (n=218)** |
| --- | --- | --- | --- | --- | --- |
| **Demographics** | | | |  |  |
|  | Sex, female | | | 493 (51.2) | 98 (45.0) |
|  | Age, years, mean (SD) | | | 75.8 (6.5) | 74.3 (6.4)* |
|  | Living status, living alone | | | 405 (42.1) | 75 (34.4)* |
|  | Education | *Primary* | | 394 (41.0) | 84 (38.5) |
|  |  | *Secondary* | | 448 (46.7) | 104 (47.7) |
|  |  | *Higher* | | 118 (12.3) | 30 (13.8) |
|  | Income | *Low* | | 222 (25.1) | 39 (19.3) |
|  |  | *Moderate-low* | | 203 (22.9) | 50 (24.8) |
|  |  | *Moderate-high* | | 256 (28.9) | 57 (28.2) |
|  |  | *High* | | 204 (23.1) | 56 (27.7) |
|  | Retired | | | 893 (95.9) | 202 (92.7)* |
| **Lifestyle** | | | |  |  |
|  | Current smoking | | | 195 (20.3) | 27 (12.4)* |
|  | Alcohol, units/week, median (IQR) | | | 3 (0−12) | 3 (0−7) |
|  | LAPAQ | *Quartile 1* | | 270 (28.1) | 59 (27.1) |
|  |  | *Quartile 2* | | 237 (24.6) | 46 (21.1) |
|  |  | *Quartile 3* | | 237 (24.6) | 57 (26.1) |
|  |  | *Quartile 4* | | 218 (22.7) | 56 (25.7) |
| **Clinical** | | | |  |  |
|  | MMSE, median (IQR) | | | 28 (26−29) | 28 (26−29) |
|  | CES-D, median (IQR) | | | 6 (3−12) | 5 (2−10)* |
|  | Chronic diseases | *Pulmonary disease* | | 142 (14.8) | 35 (16.1) |
|  |  | *Heart disease* | | 254 (26.4) | 58 (26.6) |
|  |  | *Peripheral artery disease* | | 112 (11.7) | 16 (7.3) |
|  |  | *Diabetes* | | 77 (8.0) | 12 (5.5) |
|  |  | *CVA* | | 76 (7.9) | 11 (5.0) |
|  |  | *OA or RA* | | 448 (46.6) | 97 (44.5) |
|  |  | *Cancer* | | 115 (12.0) | 22 (10.1) |
|  | Polypharmacy | | | 166 (17.3) | 24 (11.0)* |
|  | BMI, kg/m^2^, mean (SD) | | | 26.8 (4.2) | 25.9 (3.8)* |
| **Physical performance** | | | |  |  |
|  | Cardigan test, sec, mean (SD) | | | 13.7 (6.5) | 12.9 (6.2) |
|  | 5-times STS test, sec, mean (SD) | | | 13.6 (5.4) | 12.7 (3.7)* |
|  | Gait speed, meter/sec, mean (SD) | | | 0.8 (0.3) | 0.9 (0.3)* |
| **MetS components** | | | |  |  |
|  | BP, mmHg, mean (SD) | | *Systolic* | 151.8 (26.0) | 153.6 (23.9) |
|  |  |  | *Diastolic* | 82.9 (13.5) | 82.6 (13.4) |
|  | WC, cm, mean (SD) | | *Males* | 98.8 (10.0) | 97.3 (10.0) |
|  |  |  | *Females* | 93.3 (11.7) | 87.8 (10.8)* |
|  | Triglycerides, mmol/l, mean (SD) | | | 1.5 (0.8) | 1.4 (0.7)* |
|  | HDL, mmol/l, mean (SD) | | *Males* | 1.3 (0.4) | 1.2 (0.3)* |
|  |  |  | *Females* | 1.5 (0.4) | 1.4 (0.3) |
|  | Fructosamine, µmol/L, mean (SD) | | | 232.3 (36.2) | 242.5 (37.8)* |

**S1 Table.** Characteristics of participants that were included in the longitudinal sub-cohort (n=218), and participants without measurement of MetS (n=962).

All variables are presented as n (%) unless indicated otherwise. BMI: Body Mass Index, BP: Blood Pressure, CES-D: Centre for Epidemiologic Studies-Depression Scale, CVA: Cerebrovascular Accident, HDL: High-Density Lipoprotein cholesterol, IQR: Interquartile Range, LAPAQ: LASA Physical Activity Questionnaire, MetS: Metabolic Syndrome, MMSE: Mini–Mental State Examination, N: Number of participants, OA: Osteoarthritis, RA: Rheumatoid Arthritis, SD: Standard Deviation, STS: Sit-To-Stand, WC: Waist circumference. * Statistically significant different compared to participants without measurement of MetS (p<0.05)
